# Evaluation of Precision and Accuracy of a Cattle Behavior Sensor for Monitoring Sheep in Indoor and Pasture Systems

**DOI:** 10.3390/s26041150

**Published:** 2026-02-10

**Authors:** Kassy Gomes da Silva, Aline Maki Kadoguchi, Diógenes Adriano Duarte Santana, Melody Martins Cavalcante Pereira, Cristina Santos Sotomaior, Ruan Rolnei Daros

**Affiliations:** 1 Graduate Program in Animal Science, Pontifícia Universidade Católica do Paraná, Curitiba 80215-901, Brazil; kassy.silva@pucpr.edu.br (K.G.d.S.); aline.maki@pucpr.edu.br (A.M.K.); diogenes.adriano@pucpr.br (D.A.D.S.); melody.pereira@pucpr.br (M.M.C.P.); cristina.sotomaior@pucpr.br (C.S.S.); 2 EthoLab—Applied Ethology and Animal Welfare Lab, Pontifícia Universidade Católica do Paraná, Curitiba 80215-901, Brazil

**Keywords:** feeding behavior, collar, technology, ewes

## Abstract

The use of sensors applied to precision livestock farming is widespread in many farm species, especially dairy cattle, but there is a dearth of sensors validated for sheep farming. This study aims to validate a dairy cattle sensor collar to assess sheep ingestion, rumination, and other behaviors in two housing conditions: indoor housed and pasture. Twenty crossbred ewes were continuously monitored for 24 h per system, with video recordings analyzed by trained observers to quantify ingestion, rumination, and other behaviors. Precision (r, R^2^, Bland–Altman) and accuracy (CCC, regression slope) analyses were undertaken to assess sensor performance. The intra-rater reliability of behavior scoring was good (Kappa = 0.84, *p* < 0.01). In the indoor experiment, ingestion and rumination behaviors showed high precision (r = 0.92 and 0.79, respectively), while only ingestion time was considered accurate (CCC = 0.91). In the outdoor system, ingestion time showed moderate precision (r = 0.83) and accuracy (CCC = 0.80), whereas rumination and other behaviors presented low agreement with visual observations. The findings suggest that, while current sensors can be used to monitor sheep feeding behavior in confined environments, further refinement in algorithm and collar design is needed for effective application in grazing conditions.

## 1. Introduction

Precision livestock farming is a technology-driven approach that leverages real-time data collection and analysis to enhance the management of livestock. By using tools such as sensors and Internet of Things systems, it allows for the continuous monitoring of individual animals to optimize their health, welfare, and productivity while minimizing environmental impacts [1]. Furthermore, precision livestock farming integrates interdisciplinary expertise [2].

Sheep exhibit flock behavior and, as prey animals, tend to be discreet in showing signs of discomfort or illness. Healthy sheep mainly exhibit behaviors such as feeding and rumination [3]. Other normal behaviors include drinking water, lying down, investigating people and objects, and interacting with other members of the flock. To maintain a healthy flock, farmers must learn to recognize these changes indicative of health issues in the animals. The use of sensors helps even experienced producers to consider the benefits of data collection in decision-making processes; as well, they are already used for cattle herds [4].

Therefore, studies on animal behavior, health assessment, and animal welfare have advantages when implemented with precision technology [5]. Commercially in Brazil, there are companies providing sensors for cattle; however, there are no specific commercial products for use with sheep. Studies highlight the possibility of using triaxial accelerometers positioned on the sheep’s neck to identify resting, grazing, and walking movements [6,7]. To ensure the applicability of these technologies, validation studies are necessary. Gold standards are used to compare the results obtained with the equipment and are considered valid when precision and accuracy are satisfactory [8].

Thus, the aim of this study was to test equipment available for cattle to monitor sheep behavior, to identify the precision and accuracy of the device for this species, in two systems: indoor and pasture.

## 2. Materials and Methods

### 2.1. Collars and Video

For sensor validation, sheep behavior observation techniques were used through recordings by camera (Intelbras Im3, São José, Brazil). The experiment was conducted from November 2023 to September 2024, at the sheep facility of the Gralha Azul Experimental Farm, located in the municipality of Fazenda Rio Grande, Paraná, Brazil.

**Figure 1:**
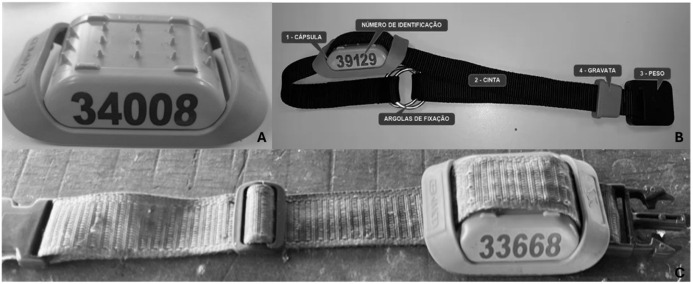
Adaptation of cattle collar for sheep. (**A**). Sensor; (**B**). Collar for cattle (Cowmed, Santa Maria, Brazil); (**C**). Adapted collar for sheep.

The collars with sensors (Cowmed, Santa Maria, Brazil) had to be adapted for sheep, as they were designed and previously validated for cattle [8]. The sensor (accelerometer), with 11.5 cm × 7 cm × 3.3 cm (Figure 1A), was removed from the original collar (Figure 1B) and placed in an adapted collar (Figure 1C), made of 100% polypropylene, with a width of 35 mm and a length of 60 cm. This material is lighter (total weight: 270 g) than the original collar for cattle (total weight: 500 g). At the moment of animal collar installation, the wool around the neck was manually parted to achieve closer contact with the skin. This physical adaptation of the collar was the sole modification performed. No species-specific calibration or adjustment of accelerometer thresholds were performed; the device was evaluated in its original factory configuration developed for cattle, as these proprietary specifications were not disclosed by the manufacturer. The sensors were synchronized to GMT-03. A receiver antenna, from the same company, was used to collect data from the sensor (wireless). The sensor records data by minute and converts it into 1 h intervals, with the number of minutes of each behavior per hour. The sensor was developed to assess three major behaviors (rumination, ingestion, and other behaviors) [8].

Given the necessity of establishing a robust gold standard for the validation of behavioral monitoring technologies [9], two observers were trained to observe the video datasets corresponding to the time the animals spent in each of the following actions: rumination (defined as the return of ruminal content to the mouth, followed by remastication and swallowing), ingestion (defined as taking feed from the feeder trough with the head directed towards it, chewing or ingesting feed, and grazing at the time of observation, along with water intake observation), and other behaviors (defined as activities such as walking, defecating, urinating, being still, moving, running, and interacting with other sheep). These groups of behaviors are the ones described by the manufacturer. To test the intra-rater reliability, a visual observation of 60 min from two live animals, each 1 min, was scored for their behaviors (i.e., ingestion, ruminating, other behaviors) [10,11]. The videos were analyzed using the BORIS software version v. 8.22.16 (https://www.boris.unito.it/ (accessed on 10 January 2024)) and Excel spreadsheets, where behaviors and their respective durations were recorded. Intra-rater reliability was tested using the Kappa coefficient with the R package ‘irr’, version 4.4.1. Results for the Kappa coefficient were interpreted according to Schober et al. [12], where values > 0.85 indicated excellent agreement, 0.70 to 0.84 indicated good agreement, 0.55 to 0.69 indicated moderate agreement, and <0.54 indicated weak and poor agreement.

### 2.2. Experiment 1: Indoor Housing

For data collection, observations of sheep indoor were conducted. The sample consisted of 20 adult crossbred non-lactating ewes (Texel, Ile de France, or Suffolk) during summer season. Sensor validation was based on video recordings collected over several days during daylight hours, accumulating 24 h of footage per sheep (20 sheep total), for a total of 480 h of video data. A fixed camera positioned in the ceiling was used for video recordings. The animals were housed as a single group in a collective pen (1 m^2^ per animal) within a shelter featuring lateral openings and a wooden structure, with access to water, grass hay, silage, and commercial feed. To ensure accurate individual identification for correlating behavioral observations with sensor data, each sheep was clearly marked on its back (fleece) with a unique color pattern using a livestock marking stick. When not under experiment, the flocks were always released for half of the day.

### 2.3. Experiment 2: Outdoor Housing

The management protocol restricted pasture access to daylight hours (7:00 AM–5:00 PM), after which the animals were moved indoors and housed in a pen overnight. Consequently, all video observations were conducted exclusively within this daytime grazing period. The same animals used for experiment 1 were used in the second experiment, conducted during the winter season. For this experiment, video recordings were conducted on small groups of 4 sheep. To ensure clear visibility, the available paddock area for each group was temporarily restricted, allowing all 4 animals to be captured within the field of view of a single fixed camera. The camera was positioned at the perimeter of this restricted area to optimize visualization and accurate behavioral classification. The height of oat pasture varied from 10 cm to 40 cm, with a stocking density of 12.5 m^2^ per animal. A total of 24 h of video recording was conducted for each sheep, representing a cumulative total of data collected over multiple days, excluding the overnight indoor housing period. The animals had access to water and silage in the paddock.

### 2.4. Statistical Analysis

The R program 4.4.1 was used, with the follow R packages: ‘blandr’, ‘ggplot2’, ‘lme4’, ‘lmerTest’, ‘sjPlot’, ‘ggpubr’, and ‘MuMIn’. To compare the data provided by the sensor and visual observation, the statistical analysis included the evaluation of the accuracy and precision of behaviors (rumination, ingestion, and other behaviors), based on the study by Grinter et al. [13]. The experimental unit used was sheep/24 h of video. To assess precision, Pearson’s correlation test (r), the coefficient of determination (R^2^), and the Bland–Altman plot were used. To be considered precise, the correlation value had to be greater than 0.70, R^2^ > 0.70, and the Bland–Altman mean bias had to include zero within the 95% confidence interval (CI). To assess accuracy, Mixed Linear Regression tests and the Concordance Correlation Coefficient (CCC) were used. To be considered accurate, the evaluated behavior had to have a CCC > 0.90 and a regression slope not significantly different from 1.00.

## 3. Results

The intra-rater reliability of behavior scoring was tested and found to be good (Kappa = 0.84, *p* < 0.01).

### 3.1. Indoor Housing Experiment

#### 3.1.1. Precision

**Figure 2:**
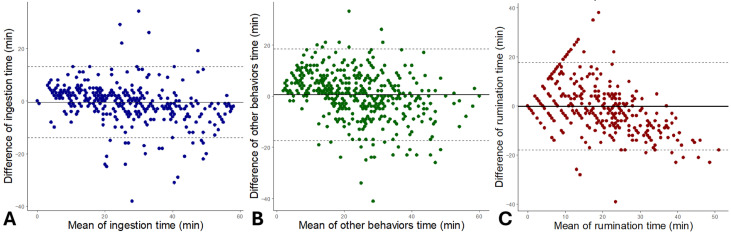
Agreement between the sensor measurements and visual observations of ingestion time (**A**), other behaviors time (**B**), and rumination time (**C**) displayed in Bland–Altman plots (continuous line indicates bias; dashed lines indicate upper and lower limits of agreement (95% CI)) of sheep behavior in indoor housing.

The r was 0.92, 0.80, and 0.79 for ingestion time, other behaviors time, and rumination time, respectively. The R^2^ value was 0.87, 0.72, and 0.69 for ingestion time, other behaviors time, and rumination time, respectively. For each behavior, Bland–Altman plots were used to assess the differences between the collar and visual observations (Figure 2). All of them had an agreement between the sensor and visual observation. The mean of ingestion time was −0.43 min (CI: −13.88 to 13.02); for rumination time, it was −0.13 min (CI: −18.07 to 17.80); and other behaviors time was 0.54 min (CI: −17.27 to 18.35).

#### 3.1.2. Accuracy

**Figure 3:**
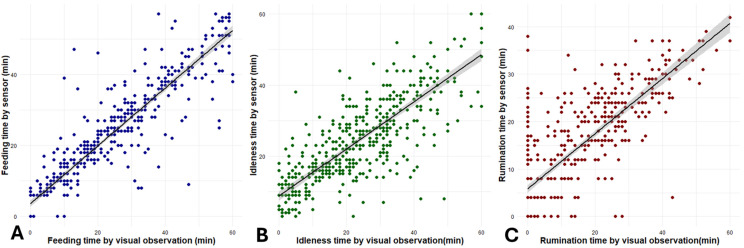
Regression slopes of ingestion (**A**), other behaviors (**B**), and rumination (**C**), comparing the sensor (y-axis) with visual observations (x-axis), of sheep behavior in indoor housing.

The CCC value was 0.91, 0.79, and 0.75 for ingestion time, other behaviors time, and rumination time, respectively. Only ingestion time achieved 0.90, allowing it to be considered accurate. The slope of regression was found to be 1.04 (CI: 1.00–1.08) for ingestion time, 0.88 (CI: 0.82–0.92) for other behaviors time, and 1.10 (CI: 1.03–1.17) for rumination time (Figure 3). Only ingestion time had a slope not different than 1.00.

### 3.2. Outdoor Housing Experiment

#### 3.2.1. Precision

**Figure 4:**
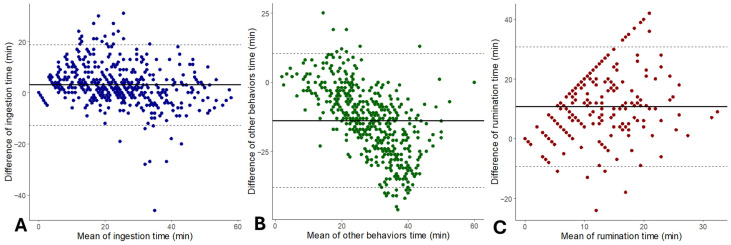
Agreement between the sensor measurements and visual observations of ingestion time (**A**), other behaviors time (**B**), and rumination time (**C**) displayed in Bland–Altman plots (continuous line indicates bias; dashed lines indicate upper and lower limits of agreement (95% CI)) of sheep behavior in pasture.

The r was of 0.83, 0.48, and 0.29 for ingestion time, other behaviors time, and rumination time, respectively. The R^2^ value was 0.80, 0.46, and 0.49 for ingestion time, other behaviors time and rumination time, respectively. Bland–Altman plots showing differences between the collar and visual observations are presented in Figure 4. The sensor overestimated ingestion time (+3.07 min/h; CI: 2.34 to 3.79) and rumination time (+10.79 min/h; CI: 9.87 to 11.70) compared to visual observations. Other behaviors time was underestimated by the sensor (−13.80 min/h; CI: −14.91 to −12.69).

#### 3.2.2. Accuracy

**Figure 5:**
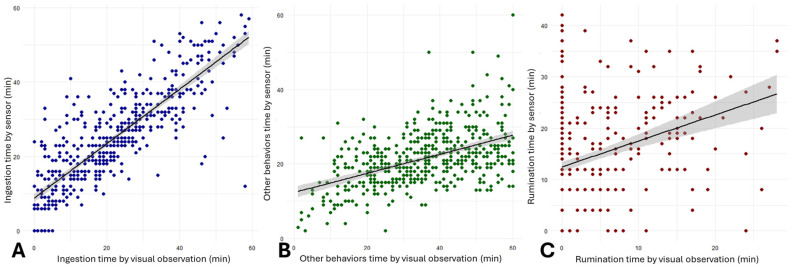
Regression slopes of ingestion (**A**), other behaviors (**B**), and rumination (**C**), comparing the sensor (y-axis) with visual observations (x-axis) of sheep behavior in pasture.

The CCC value was 0.80, 0.23, and 0.14 for ingestion time, other behaviors time, and rumination time, respectively. None of them achieved 0.90, which would be considered accurate. The slope of regression was found to be 0.95 (IC: 0.90–1.00) for ingestion time, 0.83 (IC: 0.70–0.97) for other behaviors time, and 0.22 (IC: 0.17–0.26) for rumination time (Figure 5).

### 3.3. General Results

For indoor housing, the behaviors of ingestion and rumination were considered precise, but only ingestion time was considered accurate (Table 1). In the second experiment (grazing outdoors), although there were some positive results for ingestion time regarding precision and accuracy, none of the behaviors can overall be deemed precise or accurate when considering all evaluations (Table 1).

## 4. Discussion

This study evaluated the application of a cow behavior sensor available commercially in sheep in two different situations: indoor and pasture. Testing commercially available products in new situations can enable the application of technologies already known. Thus, instead of investing in the development of technology from the ground up, an investment in updating existing equipment and algorithms may be sufficient to reach new markets. Overall, our findings indicate that this commercial sensor developed to be applied in dairy cattle farming has potential to be implemented to monitor behaviors of indoor housed sheep. Although originally validated in cattle [8], the suitability of a 1 min observation interval for sheep arises from the comparable temporal structure of the targeted behaviors, as ingestion and rumination in both species are long-duration states that extend well beyond 1 minute.

For use in indoor housing, the sensor showed precision and accuracy for ingestion time. Ruminating time has shown variation according to sensor position and its settings [14]. According to the manufacturer’s instructions, the sensor needs to be positioned in the left part of the cow’s neck. To maintain its position, a counterweight (240 g) is used to fit the collar of the neck of the cow. With the adaptation for sheep, to reduce collar weight, this counterweight was removed, and a plastic slider adjuster was used to fit the collar to the sheep’s neck. Although the collar was correctly positioned in the left part of the neck, in some cases, it rotated and changed its position to the middle of the neck. This occurred in both indoor and outdoor environment types, which could interfere mainly in ruminating time data, because rumination movements could not be correctly detected with the change in collar position. The accelerometer records variations in acceleration; thus, the periodic and repetitive head and jaw movements during rumination can be detected. Currently, there are no commercially available sensors for detecting rumination in sheep [15]. An adaptation of the collar to methods of preventing its movement could be tested to avoid its interference. Since other behaviors time was defined as any behavior other than ingestion and ruminating, inaccurate measurements of ruminating time could directly affect the calculation of other behaviors time. Therefore, with improved precision in measuring ruminating time, we can expect an improvement in the accuracy of other behaviors time as well. A similar situation was reported by Lovatti et al. [8], who noted that the algorithm may have misclassified other behaviors as one of the behaviors of interest in their study. The authors emphasized that the ethogram used in their investigation might differ from the one employed in the development of the sensor algorithm. A comparable scenario may have occurred in the present study, as the algorithm was trained for cattle but applied to sheep. Thus, an option for enhancing sheep sensors is the incorporation of machine learning algorithms into behavioral monitoring systems. Mansbridge et al. [16], utilizing accelerometers and gyroscopes to collect data from collars and ear tags on sheep, tested different machine learning algorithms. The behaviors of ingestion, rumination, and non-ingestion activities (considered other behaviors in the current study) were accurately identified, particularly with the use of the “Random Forest” algorithm.

The pasture evaluation showed a higher ingestion frequency recorded by the sensor compared to visual observation. As described by Iqbal et al. [17], cows’ foraging movements in pasture, which may appear as if they are grazing but are actually searching for food, can impair the collar’s algorithm. The same may have occurred with the sheep in this study, as, being selective grazers [18], they spent part of their time in pasture searching for grass, mimicking ingestion movements without any ingestion of feed.

For rumination time, an important factor to consider is the volume of wool between the sensor and the sheep’s skin, considering that the layer of wool keeps the sensor away from the center of mass and that devices not fixed to the animal may result in less accurate acceleration measurements [19]. In the indoor housing, which took place in summer, the animals had recently been shorn, increasing the contact area between the skin and the sensor. In contrast, the outdoor sheep group had more wool. The presence of wool during winter may have hindered and confounded our results for the outdoor trial; therefore, we acknowledge that such a limitation needs to be considered when interpreting our findings. Future studies are planned to overcome this limitation. Although efforts were made to part the wool as much as possible to expose the neck to the collar, it is possible that, due to the animals’ movement, some wool returned to the area and reduced the contact between the skin and the collar. In this case, shearing the area may prevent the issue. However, studies to determine the optimal wool length and the interval between shearings are important to assess the feasibility of application by sheep producers.

## 5. Conclusions

In indoor housing, the sensor showed ingestion time precisely and accurately for adult sheep. Although ruminating time was considered precise, improvements are suggested to achieve adequate accuracy for ruminating and other behaviors. For pasture or when sheep are not sheared, new experiments to test species-specific collar improvements are suggested.

## Figures and Tables

**Table 1:** Precision and accuracy results between sensor and visual observation, for ingestion, rumination, and other behaviors, for indoor and pasture experiments.

	Indoor			Outdoor (Pasture)		
	Ingestion	Other Behaviors	Rumination	Ingestion	Other Behaviors	Rumination
Precision ^1^						
r	Yes	Yes	Yes	Yes	No	No
R^2^	Yes	No	Yes	Yes	No	No
Bland–Altman	Yes	Yes	Yes	No	No	No
Is precise?	Yes	No	Yes	No	No	No
Accuracy ^2^						
CCC	Yes	No	No	No	No	No
Slope of regression	Yes	No	No	Yes	No	No
Is accurate?	Yes	No	No	No	No	No

^1^ To be considered precise, the correlation value had to be greater than 0.70, R^2^ > 0.70, and the Bland–Altman mean bias had to include zero within the 95% confidence interval. ^2^ To be considered accurate, the evaluated behavior had to have a CCC > 0.90 and a regression slope not significantly different from 1.00.

## Data Availability

Data available on request.
